# Supplementary material for: Isolation, identification and antibiotic resistance profile of thermophilic Campylobacter species from Bovine, Knives and personnel at Jimma Town Abattoir, Ethiopia
Source: PLoS One. 2022 Oct 21;17(10):e0276625. doi: 10.1371/journal.pone.0276625 (PMC9586361; doi:10.1371/journal.pone.0276625)
Supplement: S1 File — (DOCX) [file pone.0276625.s001.docx]

# Procedures or Protocols

# Annex 1: Selective media: Selective media for isolation:

Many media can be used in the recovery of Campylobacter species. Modified charcoal, Cefoperazone, Deoxycholate agar (mCCDA), is the recommended medium, although alternative media may be used. A detailed description on Campylobacter detection by culture and the variety of existing media (OIE. 2008). The selective media can be divided into two main groups: blood-containing media and charcoal-containing media. Blood components and charcoal serve to remove toxic oxygen derivatives. Most media are commercially available. The selectivity of the media is determined by the antibiotics used. Cephalosporin (generally Cefoperazone)are used, sometimes in combination with other antibiotics (e.g. vancomycin, trimethoprim). Cycloheximide (actidione) and more recently amphotericin B are used to inhibit yeasts and molds (OIE, 2008). The main difference between the media is the degree of inhibition of contaminating flora. All the selective agents allow the growth of both C. jejuni and C. coli. There is no medium available that allows growth of C. jejuni and inhibits C. coli or vice versa. To some extent, other Campylobacter species (e.g. C. lari, C. upsaliensis, C. helveticus, C. fetus and C. hyointestinalis) will grow on most media, especially at the less selective temperature of 37°C.

**Incubation:** Atmosphere: Micro aerobic atmospheres of 5–10% oxygen, 5–10% carbon dioxide are required for optimal growth (OIE, 2OO8). Appropriate atmospheric conditions may be produced by a variety of methods. In some laboratories, (repeated) gas jar evacuations followed by atmosphere replacement with bottled gasses are used. Gas generator kits are available from commercial sources. Variable atmosphere incubators are more suitable if large numbers of cultures are undertaken.

**Temperature:** Media may be incubated at 37°C or 42°C, but it is common practice to incubate at 42°C to minimize growth of contaminants and to select for optimal growth of C. jejuni/C. coli. The fungistatic agents Cycloheximide or amphotericin are added in order to prevent growth of yeasts and mold at 37°C (OIE, 2008).

**Time:** Campylobacter jejuni and C. coli usually show growth on solid media within 24–48 hours at 42°C. As the additional number of positive samples obtained by prolonged incubation is very low, 48 hours of incubation is recommended for routine diagnosis (OIE, 2008).

**Confirmation**: A pure culture is required for confirmatory tests, but a preliminary confirmation can be obtained by direct microscopic examination of suspect colony material.

1. Identification on solid medium: On Skirrow or other blood-containing agar, characteristic Campylobacter colonies are slightly pink, round, convex, smooth and shiny, with a regular edge. On charcoal-based media such as mCCDA, the characteristic colonies are greyish, flat and moistened, with a tendency to spread, and may have a metal sheen.
2. Microscopic examination of morphology and motility: material from a suspect colony is suspended in saline and evaluated, preferably by a phase-contrast microscope, for characteristic, spiral or curved slender rods with a corkscrew-like motility. Older cultures show less motile coccoïd forms.
3. Detection of Oxidase: take material from a suspect colony and place it on to a filter paper moistened with Oxidase reagent. The appearance of a violet or deep blue color within 10 seconds is a positive reaction. If a commercially available Oxidase test kit is used, follow the manufacturer’s instructions.
4. Micro aerobic growth at 25°C: Inoculate the pure culture on to a non-selective blood agar plate and incubate at 25°C in a Micro aerobic atmosphere for 48 hours.
5. Aerobic growth at 41.5°C: Inoculate the pure culture on to a non-selective blood agar plate and incubate at 41.5°C in an aerobic atmosphere for 48 hours.
6. Latex agglutination tests for confirmation of pure cultures of C. jejuni/C. coli (often also including C. lari) are commercially available.

## Annex 2: Gram stain procedures

1. Using a sterile inoculating loop, add 1 drop of sterile water to the slide. Prepare a mixed smear of culture.
2. Air dry and Heat fix.
3. Cover the smear with Crystal Violet (primary stain) for 1 min.
4. Gently wash off the slide with water.
5. Add Gram’s Iodine (mordant) for 1 min.
6. Wash with water.
7. Decolorize with 95% ethanol. This is the "tricky" step. Stop decolorizing with alcohol as soon as the purple colour has stopped leaching off the slide (time will vary depending on thickness of smear). Immediately wash with water. Be sure to dispose of all ethanol waste in the appropriately labeled waste container.
8. Cover the smear with Safranin for 30 seconds.
9. Wash both the top and the bottom of the slide with water.
10. Blot the slide with bibulous paper.
11. Using the 10x objective lens and using the 100x (oil immersion lens), focus first on the line and then on the smear.

Interpretation: Bluish purple colour indicates gram-positive and pinkish colour indicate gram-negative bacteria.

# Annex 3: Catalase test procedures

1. Pick a colony from an48-72 hour’s culture and place it on a clean glass slide.
2. Put one drop of 3% H_2_O_2_ over the organism on the slide.
3. Observe for immediate bubbling (gas liberation) and record the result.

Interpretation: A positive result is the rapid evolution of O_2_ as evidenced by bubbling and a negative result is no bubbles or only a few scattered bubbles.

# Annex 4: Oxidase test procedure

1. Take a filter paper soaked with the substrate tetramethyl-p-phenylenediamine dihydrochloride.
2. Moisten the paper with sterile distilled water.
3. Pick the colony to be tested with wooden or platinum loop and smear in the filter paper.
4. Observe inoculated area of paper for a color change to deep blue or purple within 10-30 second.

Interpretation: Positive test: Development of deep purple color within 10 seconds

Negative test: Absence of color

# Annex 5: Typical biochemical reaction of commonly isolated of thermotolerant campylobacter

Table 1. Typical biochemical reaction of commonly isolated of thermotolerant campylobacter

| **Characteristic** | ***C.jejuni*** | ***C.coli*** | ***C.lari*** | ***C.upsaliensis*** |
| --- | --- | --- | --- | --- |
| **Growth at 25^0^C** | - | - | - | - |
| **H_2_S production** | - | (+) | - | - |
| **Nalidixic acid** | S | S | R | S |
| **Cephalothin** | R | R | R | S |
| **Catalase test** | + | + | + | -or slight |
| **Hippurate hydrolysis** | + | - | - | - |

(+) = slightly positive; S = sensitive; R = resistant.

a) Growth at 25°C and 42°C

A cell-suspension (~McFarland no. 1) is inoculated onto two blood-based medium-plates. Each plate is incubated under the specified atmospheric conditions at 25°C and 42°C. Control strains are tested in parallel.

b) Oxidase and Catalase: Tests are performed according to a standard bacteriological protocol. Control strains are tested in parallel.

c) Hydrogen sulphide (H_2_S) production in TSI medium

This hydrogen sulphide (H_2_S) test is done on triple sugar iron agar (TSI) under the specified growth conditions. The medium contains peptone (20 g/liter), meat extract (2.5 g/liter), yeast extract (3 g/liter), sodium chloride (5 g/liter), ferric citrate (0.5 g/liter), sodium thiosulphate (Na_2_S_2_O_3_) (0.5 g/liter), lactose (10 g/liter), sucrose (10 g/liter), glucose (1 g/liter), phenol red (0.024 g/liter), agar (11 g/liter), and distilled water (to 1 liter). These mediums are sterilized after distribution into tubes by autoclaving at 115°C for 15 minutes and are solidified to obtain a slope. A cell suspension (McFarland standard) is inoculated onto the slope and into the medium by a loop. A color change from red to black indicates H_2_S production. Control strains are tested in parallel.

d) Sensitivity to Cephalothin and Nalidixic acid

Sensitivity to Cephalothin (CN) and Nalidixic acid (NA) is tested by the disks containing CN (30 µg) or NA (30 µg). For the test, 72-hour cultures are suspended in PBS at a concentration of10^9^ bacteria/ml. The culture medium is dried before the culture is deposited on the surface. Using the suspension, 100 µl are spread onto the basic blood medium. The sensitivity disks are then placed on top. These plates are incubated at 37°C in the specified atmosphere (see Section B.1.d.ii), and examined after 48 hours and 72 hours. A zone of inhibition of at least 3 mm around a disk indicates that the strain is sensitive to this antibiotic (OIE, 2008).

e)Hippurate Hydrolysis Test

- Suspend sufficient bacterial cells in 0.8 ml sterile distilled water in a small tube to form a milky suspension. Add 0.2 ml of a 5% (5 g/100 ml) sodium Hippurate aqueous solution and incubate at 37°C for 4 h.
- Add 0.4 ml of ninhydrin solution (3.5 g in 100 ml of a 1:1 (v:v) acetone and butanol mixture). Do not shake tubes after ninhydrin addition. Incubate for up to 20 min. at room temperature.
- Hydrolysis of Hippurate is indicated by a colour change to dark purple due to the release of glycine. A purple colour is a positive result. No colour or a faint trace of purple is a negative result.

# Annex 6: Procedure for performing the disc diffusion test

Inoculums Preparation

The growth method is performed as follows

1. At least three to five well-isolated colonies of the same morphological type are selected from an agar plate culture. The top of each colony is touched with a loop, and the growth is transferred into a tube containing 4 to 5 ml of a suitable broth medium, such as tryptic soy broth.

2. The broth culture is incubated micro aerobicallyat 37°C and 42°C until it achieves or exceeds the turbidity of the 0.5 McFarland standards (usually 2 to 6 hours)

3. The turbidity of the actively growing broth culture is adjusted with sterile saline or broth to obtain turbidity optically comparable to that of the 0.5 McFarland standards. This results in a suspension containing approximately 1 to 2 x 10^8^ CFU/ml for *E.coli* ATCC 25922. To perform this step properly, either a photo-metric device can be used or, if done visually, adequate light is needed to visually compare the inoculums tube and the 0.5 McFarland standard against a card with a white background and contrasting black lines.

Inoculation of Test Plates

1. Optimally, within 15 minutes after adjusting the turbidity of the inoculums suspension, a sterile cotton swab is dipped into the adjusted suspension. The swab should be rotated several times and pressed firmly on the inside wall of the tube above the fluid level. This will remove excess inoculums from the swab.

2. The dried surface of a Mueller-Hinton agar plate is inoculated by streaking the swab over the entire sterile agar surface. This procedure is repeated by streaking two more times, rotating the plate approximately 60° each time to ensure an even distribution of inoculums. As a final step, the rim of the agar is swabbed.

3. The lid may be left ajar for 3 to 5 minutes, but no more than 15 minutes, to allow for any excess surface moisture to be absorbed before applying the drug impregnated disks.

NOTE: Extremes in inoculums density must be avoided. Never use undiluted overnight broth cultures or other nonstandard inoculation for streaking plates.

Application of Discs to Inoculated Agar Plates

1. The predetermined battery of antimicrobial discs is dispensed onto the surface of the inoculated agar plate. Each disc must be pressed down to ensure complete contact with the agar surface. Whether the discs are placed individually or with a dispensing apparatus, they must be distributed evenly so that they are no closer than 24 mm from centre to centre. Ordinarily, no more than 12 discs should be placed on one 150 mm plate or more than 5 discs on a 100 mm plate. Because some of the drug diffuses almost instantaneously, a disc should not be relocated once it has come into contact with the agar surface. Instead, place a new disc in another location on the agar.
2. The plates are inverted and placed in an incubator at 42°C for 48 hours in anaerobic jar using CO_2_ generating kits.

Reading Plates and Interpreting Results

1. After 44 to 48 hours of incubation, each plate is examined. If the plate was satisfactorily streaked, and the inoculums were correct, the resulting zones of inhibition will be uniformly circular and there will be a confluent lawn of growth. If individual colonies are apparent, the inoculum was too light and the test must be repeated. The diameters of the zones of complete inhibition (as judged by the unaided eye) are measured, including the diameter of the disc. Zones are measured to the nearest whole millimetre, using sliding callipers or a ruler, which is held on the back of the inverted petriplate.
2. The zone margin should be taken as the area showing no obvious, visible growth that can be detected with the unaided eye. Faint growth of tiny colonies, which can be detected only with a magnifying lens at the edge of the zone of inhibited growth, is ignored. However, discrete colonies growing within a clear zone of inhibition should be sub-cultured, re-identified, and retested. Strains of *Proteus* spp. may swarm into areas of inhibited growth around certain antimicrobial agents. With *Proteus* spp., the thin veil of swarming growth in an otherwise obvious zone of inhibition should be ignored. With trimethoprim and the sulphonamides, antagonists in the medium may allow some slight growth; therefore, disregard slight growth (20% or less of the lawn of growth), and measure the more obvious margin to determine the zone diameter.
3. The sizes of the zones of inhibition are interpreted by referring to Tables 2A through 2I (Zone Diameter Interpretative Standards and equivalent Minimum Inhibitory Concentration Breakpoints) of the NCCLS, 2002: Performance Standards for Antimicrobial Susceptibility Testing: Twelfth Informational Supplement and the organisms are reported as susceptible, intermediate, or resistant to the agents that have been tested. Some agents may only be reported as susceptible, since only susceptible breakpoints are given.

Table 2. Interpretation/break points for antimicrobial susceptibility pattern of Campylobacter species isolated from live bovine to abattoir in Jimma town, October to September 2020.

| Selected Antibiotics | | Code | Disk concentration (μg) | Zone diameter break point(mm) | | |
| --- | --- | --- | --- | --- | --- | --- |
|  |  |  |  | S ≥ | I | R < |
| Quinolone and Fluoroquinolones | Ciprofloxacin | CIP | 10 | 21 | 16-20 | 15 |
|  | Nalidixic acid | NA | 30 | 19 | 14-18 | 13 |
| Lincosamides | clindamycin | DA | 10 | 20 | - | 19 |
| Phenicol | Chloramphenicol | C | 30 | 18 | 13-17 | 12 |
| Tetracycline | Tetracycline | TE | 30 | 19 | 15-18 | 14 |
| Penicillin | Ampicillin | AMP | 10 | 17 | 14-16 | 13 |
| Cephalosporin | Cephalothin | Ch | 30 | 18 | 15-17 | 14 |
|  | Ceftriaxone  Cefixime  Cefotaxime | CRO  CFM  CTX | 30  5  30 | 23  20  30 | 20-22  -  - | 19  19  29 |
| Sulfonamides | Sulphamethazole trimethoprim | SXT | 25 | 14 | - | 13 |
