# Supplementary material for: Isolation, identification and antibiotic resistance profile of thermophilic Campylobacter species from Bovine, Knives and personnel at Jimma Town Abattoir, Ethiopia
Source: PLoS One. 2022 Oct 21;17(10):e0276625. doi: 10.1371/journal.pone.0276625 (PMC9586361; doi:10.1371/journal.pone.0276625)
Supplement: Attachment — Submitted filename: comments to authors.docx [file pone.0276625.s002.docx]

**Comments to the Author:**

The paper described the “Isolation, identification and antibiogram profile of thermophilic Campylobacter species from bovine, knives and personnel at Jimma Town abattoir, Ethiopia”.

**Major comment:** After carefully reading this paper, it was observed that the findings of the paper are of local interest and importance. Other readers may not be benefited by the data. Further, the study carried out at only one abattoir and therefore the limited number of isolates (n=38) obtained in this study are not sufficiently addressed to draw any logistic conclusion. The article does not have any novelty of data or any molecular characterization of isolates. Further, the data of the study is also not presented well in the manuscript.

Keeping in view of the facts, the article may not be found suitable for publication in the reputed and high impact Plos One journal.

XXXXXXXX

**Minor comments :**

The manuscript presents the research to understand the current status of Antimicrobial Resistance (AMR) in Campylobacter isolates from faecal samples of bovine, carcases of slaughtered animals, knives and personnel employed at abattoir.

Overall, the manuscript requires editing on the grammar at many places to improve the clarity and data presentation. Some sentences are not clear and difficult to understand. Objective to rephrase, not clear, and to show novelity or improvement from previous studies carried in this era.

In Methodology, what i am missing is the absence of molecular study for identification of different species and for detection of different AMR and Virulence genes.
Emphasize the new and important aspects of your study and put your findings in the context of the totality of the relevant evidence.
I didn’t find any standard strains of campylobacter( be it jejuni, coli, lari) were used for this study?

As a general comment, no limitations of the study were given in manuscript.  In my mind the major limitation is the absence of genotypic studies for AMR and virulence of isolates it could have made your study more meaningful and interesting.
The objective of this  manuscript was to estimate the occurrence and antimicrobial resistance of thermophilic Campylobacter strains isolated from from faecal samples of bovine, carcases of slaughtered animals, knives and personnel employed at abattoir in Ethiopia. There are many articles with similar objectives and I do not observe in this any original element beyond the region studied. However, this fact seems to me not to be enough and perhaps the authors should consider a journal with local impact.
