# Supplementary material for: Isolation, identification and antibiotic resistance profile of thermophilic Campylobacter species from Bovine, Knives and personnel at Jimma Town Abattoir, Ethiopia
Source: PLoS One. 2022 Oct 21;17(10):e0276625. doi: 10.1371/journal.pone.0276625 (PMC9586361; doi:10.1371/journal.pone.0276625)
Supplement: Attachment — Submitted filename: Response to Reviewers.docx [file pone.0276625.s004.docx]

Rebuttal Letter

Reviewer #1

1. We thank the reviewer for the fruitful comments

Major comment:

a. We the authors agree with the reviewer comment that it was better if the study was done at national level. However, Jimma Town is not as such small town and it is home for more than 207,573 (projected population in 2021/not actual) with 50.52km^2^ in south-western Ethiopia. There is only one abattoir in the town delivering service for the vast population in the town. The research finding will benefit the local and national government to take measures in Jimma abattoir in particular and abattoirs in the country in general. Researchers/scientific community will be benefitted to prepare review articles, taking this findings as a baseline research and to consider the aggravation of antimicrobial resistance pattern both in human and animal populations. To make it very clear to the international readers, we elaborated the background of our study setting.
b. It was better if the number of isolates obtained (n=38) was increased. However, our finding is consistent (relatively high) with similar previous researches done in Ethiopia.
c. We strongly agree with the comment not to use molecular characterization as diagnostic method and we included in the study limitation. It was also our plan and tried our best to get primers from abroad, but we missed due to COVID-19 pandemic lockdown in the world.

Minor comment

a. We thank the reviewer comment to edit the grammar and sentence structure. We improved the grammar and unclear sentences in the revised manuscript.
b. We thank the reviewer for the comment ‘absence of molecular study’. We took as limitation of the study.
c. We thank the reviewer comments on the objectives. We improved/rephrased our objectives in the revised manuscript.
d. We thank the reviewer for the comment. We included the importance of our finding in the revised manuscript.
e. We thank the reviewer for the comment to use standard strain of *Campylobacter*. We could not find standard strain for Campylobacter species from the national laboratory and we have used *E. coli* (ATCC 25922) as standard and included in the limitation of the study in the revised manuscript.
f. We thank the reviewer for the comment and the revised manuscript included ‘limitations of the study’.
g. We respect the reviewer comment and strongly disagree with the idea ‘study region is not enough reason to conduct research’. There are millions of prevalence studies for the same disease in different regions and/or time frame. In previous works, prevalence as well AMR was only determined from faecal but in our case it was from faecal, carcasses, personnel hands and knife samples. Therefore, our study is not out of this fact and we believe it is very important and respected to show the gaps and identify AMR *Campylobacter* strains in the region.

Reviewer #2

a. We thank the reviewer for the comments Line 15,18 and 23. We corrected accordingly in the revised manuscript.
b. We thank the reviewer comment on study design. We trimmed the full description of sample collection methods to make it shorter previously. It is included now (the regular basis) in the revised manuscript.
c. We thank the reviewer comment for the English language. Senior professors edited the English language of our revised manuscript.

Reviewer #3

a. We thank the reviewer comment on the tittle of the manuscript. We reformulated the tittle as ‘Isolation, identification and antibiotic resistance profile of thermophilic Campylobacter species from bovine, knives and personnel at Jimma Town abattoir, Ethiopia’.
b. We thank the reviewer for the comment on the English language improvement and our revised manuscript is edited by senior professors.
c. We thank the reviewer comment on capitalizing genus name initials in the manuscript and corrected accordingly.
d. We thank the reviewer for the comment to include molecular techniques (PCR) for confirmation of Campylobacter species. We also strongly agree with the comment, and there was no Campylobacter primer in Ethiopia and difficult to get from abroad due to COVID-19 pandemic at a time. We included in the limitation of the study in the revised manuscript.
e. We appreciate and thank the reviewer for suggesting very important research findings to compare with our work. We discussed our findings comparing with suggested studies.
f. We thank the reviewer comment and the discussion part was improved in the revised manuscript.
g. We thank the reviewer for critical review and the mentioned words in line 35 & 36 were eliminated in the revised manuscript.
h. We thank the reviewer for the comment and some points were added after reference (1,2) in the revised manuscript.
i. We thank the reviewer for the comments and paragraphs in line 38-39, 81-83 and 88-89 were reformulated in the revised manuscript.
j. We thank the reviewer for the comments and words mentioned in line 93 and 96 were replaced accordingly.

Authors’ response to academic editor

We thank the academic editor for the detailed comments on the format of the manuscript and other comments.

1. The manuscript was checked according to the journal formats.
2. The funding section was revised based on the comments given in the revised manuscript.
3. The acknowledgement part is corrected in the revised manuscript.
4. Supporting files were uploaded as indicated by the academic editor.
5. The authors prefer to display tables than figure, and figure 1 is deleted from the revised manuscript.
