# Supplementary material for: Isolation, identification and antibiotic resistance profile of thermophilic Campylobacter species from Bovine, Knives and personnel at Jimma Town Abattoir, Ethiopia
Source: PLoS One. 2022 Oct 21;17(10):e0276625. doi: 10.1371/journal.pone.0276625 (PMC9586361; doi:10.1371/journal.pone.0276625)
Supplement: Attachment — Submitted filename: Reviewer comments.docx [file pone.0276625.s003.docx]

Reviewer comments :

Title reformulate the

Precise that resistance is toward antibiotics not all antimicrobials molecules.

Check the manuscript language by fluent english speaker

Check the capital letter for the genus Campylobacter “ first letter (C)” all over the manuscript.

Biochemical tests are not enough to confirm the species. It is recommended to confirm Campylobacter species by molecular techniques such as PCR.

Which is the criteria used to differentiate C. coli from C. lari??? The identification part should be improved.

The authors should compared the antibiotic prevalent findings with others data. Here you find some useful papers.

El Baaboua, A., El Maadoudi, M., Bouyahya, A., Kounnoun, A., Bougtaib, H., Belmehdi, O., Senhaji, N. S., & Abrini, J. (2021). Prevalence and antimicrobial profiling of Campylobacter spp. Isolated from meats, animal, and human feces in Northern of Morocco. *International Journal of Food Microbiology*, *349*, 109202. https://doi.org/10.1016/j.ijfoodmicro.2021.109202

Asmai, R., Karraouan, B., Es-Soucratti, K., En-Nassiri, H., Bouchrif, B., Karib, H., & Triqui, R. (2020). Prevalence and antibiotic resistance of Campylobacter coli isolated from broiler farms in the Marrakesh Safi region, Morocco. *Veterinary World*, *13*(9), 1892–1897. https://doi.org/10.14202/vetworld.2020.1892-1897

The authors should improve the discussion section, mainly the molecular mechanism of antibiotics.

Some changes should be added:

Line 35: eliminate “and before followed”

Line 36: eliminate “ respectively”

Line 36: eliminate “ bacterium”.

Line 37: add a point after reference (1,2)

Line 38-39: reformulate the paragraph fro “ the transmission until prodiet “

Line 50: replace number by one.

Line 81,82, 83: repetition of 10 nb cary-blair media… the paragraph is recommended to be reformulated.

Line 88-89: reformulate the paragraph.

Line 93: replace anaerobic by microaerobic

Line 96: replace doing by preparing.
